# Optimizing Acetabular Component Bone Ingrowth: The Wedge-Fit Bone Preparation Method

**DOI:** 10.1155/2019/9315104

**Published:** 2019-07-04

**Authors:** Dani M. Gaillard-Campbell, Thomas P. Gross

**Affiliations:** Midlands Orthopaedics & Neurosurgery, 1910 Blanding Street, Columbia, SC 29201, USA

## Abstract

We investigate the efficacy of a modified acetabular bone-preparation technique in reducing the incidence of two clinical problems identified in hip resurfacing arthroplasty. The first issue is failure due to lack of bone ingrowth into the acetabular component. The second is a newly recognized phenomenon of early cup shift. We hypothesize that these issues might be resolved by using a “wedge-fit method”, in which the component wedges into the peripheral acetabular bone rather than bottoming out and potentially toggling on the apex of the cup. Prior to November 2011, all acetabula were reamed 1 mm under and prepared with a press-fit of the porous coated acetabular component. After November 2011, we adjusted reaming by bone density. In “soft bone” (T-score <-1.0), we underreamed acetabula by 1 mm less than the outer diameter of the cup, as was previously done in all cases. For T-scores greater than -1.0, we reamed line-to-line. Additionally, we began performing an “apex relief” starting June 2012 in all cases by removing 2 mm of apex bone with a small reamer after using the largest reamer. Failure of acetabular ingrowth occurred in 0.5% of cases before the wedge-fit method and <0.1% after. Rate of cup shift was reduced from 1.1% to 0.4%. The rate of unexplained pain between 2 and 4 years postoperatively also declined significantly from 2.6% to 1.3%. Our evidence suggests that wedge-fit acetabular preparation improves initial implant stability, leading to fewer cases of early cup shift, unexplained pain, and acetabular ingrowth failure.

## 1. Background

Hip resurfacing arthroplasty (HRA) is a less common, alternative method to total hip arthroplasty (THA) for reconstructing the arthritic hip. Its popularity has waxed and waned since its introduction in the 1950s [1, 2]. In the late 1990s, McMinn and Amstutz were instrumental in the introduction of metal-on-metal (MoM) hip resurfacing [2–4]. MoM HRA grew in popularity until 2007 [5–7]; then, it lost popularity for a variety of reasons, including the identification of adverse wear-related failure (AWRF). Some HRA failure modes are unique, but many are the same as for THA. An issue for both uncemented THA and HRA is failure of bone ingrowth into the acetabular component. Its incidence is not well known, and it is difficult to diagnose unless the implant migrates.

Our initial experience with resurfacing was promising, with a 92% 10-year implant survivorship reported in young patients; this compared favorably with registry reports [8, 9]. We therefore chose to redouble our efforts with HRA rather than follow much of the orthopedic community, in which many professionals abandoned MoM bearings after the recall of two faulty systems [10]. Our strategy was to identify modes of failure and implement steady improvements to implant design and surgical techniques. We have now improved 10-year implant survivorship to 96.5% in patients below 50 [11]. In an endeavor to further improve results, we chose to modify acetabular preparation in 2012 with hopes of more stable initial fixation. We anticipated that this would lower the rate of acetabular bone ingrowth, which had prevalence of 0.5% in our database. We hypothesized that creating a “wedge fit” while preparing and implanting the acetabular component might reduce the rate of ingrowth failure. Early asymptomatic cup shift and unexplained pain seem to be related problems that have not previously been addressed in the literature. These problems are related to the quality of the initial implant press-fit [12].

Uncemented acetabular components that fail to achieve bone ingrowth rarely develop complete radiolucent lines [13]. Thus, they can be difficult to diagnose unless they shift in position. We have not found bone scans or other tests to be helpful in diagnosing bone ingrowth failure in nonshifted cups. The definitive method to diagnose a loose cup is to test it intraoperatively, but this is not appropriate in most cases. In our experience, there are two basic presentations for failure of acetabular ingrowth. The first is a dramatic spin out of the cup, usually within a few months. In the second, a patient develops persistent pain within the first 2 years postoperatively, and the acetabular component shows a cup shift over a series of standard pelvic x-rays. In our experience, most failures of cup ingrowth become evident by 2 years postoperatively. Therefore, we diagnose symptomatic patients with failure of ingrowth when the cup moves significantly between 6 weeks and 2 years. On the other hand, some patients present excellent initial outcome, but, sometimes after 2 years, they develop persistent pain and cup migration. We consider these late loosening. We suspect that some cases of unexplained pain in THA and HRA may be due to these loose or fibrous ingrown cups [14–16], which we have discovered in some rare cases during surgical exploration for the patient's pain.

We hypothesize that adjusting acetabular preparation by bone density and adding an apex relief will improve acetabular bone ingrowth, unexplained pain, and cup stability. We refer to this collective surgical technique as the “wedge-fit method”. We postulate that a wedge fit into peripheral acetabular bone prevents the component from bottoming out and toggling on the apex of the cup, thus providing more stability and better fixation. Herein, we report our clinical outcomes with this new strategy for acetabular bone preparation.

## 2. Materials and Methods

Between September 2006 and November 2011, we performed 1496 HRAs (Group 1) with the Biomet Magnum™ uncemented acetabular component using a 1 mm under ream press-fit technique in all cases. Between June 2012 and June 2016, we performed 1565 HRAs (Group 2) using the new wedge-fit technique. All patients in both groups received the Biomet Magnum acetabular component. A minimum follow-up of 2 years was available in our prospective database, with a mean follow-up rate of 94%. We compared the two preparation techniques for three endpoints: revision for failure of acetabular ingrowth, residual unexplained pain, and early component shift.

These Biomet implants are relatively stiff with 3-6 mm thick cast cobalt-chrome components with a thin layer of titanium alloy porous plasma spray for bone ingrowth fixation. For the Biomet Magnum system, the porous plasma spray coating is similar to that seen on all other Biomet titanium plasma coated implants. However, THA cups come in a wide variety of thicknesses and coatings [17]. Therefore, it is not clear how well our technique will work in THA implants; further studies are required.

**Figure 1:**
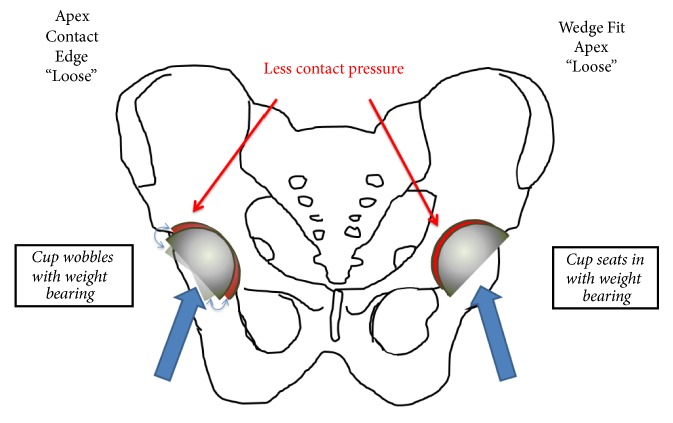
Cup fixation variation for two acetabular preparation methods.

A minimally invasive posterior approach with a 4-5-inch incision was used in most cases. In Group 1, all acetabula were serially reamed up to a size 1 mm smaller than the actual outer diameter (OD) of the component. In Group 2, we reamed bone based on the bone density as determined by DEXA scan. If the bone was hard (femoral neck T-score ≥ -1.0), the acetabulum was reamed line-to-line with the OD of the component. If the bone was soft (T-score < -1.0), the last reamer was 1 mm less than the OD of the implanted cup. In addition, 2 mm of acetabular apex bone was removed in all cases regardless of bone density by finishing with a clean reamer 5 mm smaller than the largest size. A clean reamer was used so that the amount of bone removed from the apex could be easily seen. We performed an apex relief so that the impacted component would “wedge fit” into the peripheral bone and have less chance of bottoming out on the apex (Figure 1). A metal trial component that was approximately line-to-line with the final component was placed into the reamed socket to judge if reaming depth was adequate to allow correct positioning of the component with respect to inclination, anteversion, and cup overhang. No attempt was made to judge the quality of fit with the trial component. All patients in both groups received the Biomet Magnum acetabular component.

Table 1 compares demographic data. Group 2 was slightly older, on average, with more cases of osteoarthritis and fewer cases of trauma. Because these groups are consecutive, the follow-up is not equal; however, all cases had a minimum of 2-year follow-up. Therefore, we only include failures of acetabular ingrowth diagnosed prior to 2-year follow-up for our comparison to eliminate time bias in the analysis. Since it can be difficult to diagnose failure of acetabular component ingrowth, we analyzed whether there was a difference in the rate of unexplained pain in the two groups. We defined this as cases where the pain component of the Harris hip score (HHS) [18] was less than or equal to 30 (moderate to disabling pain). We compared pain scores at the latest follow-up for both groups, but because Group 1 has a greater length of follow-up, we also compared the minimum pain score (peak amount of pain) for each patient between 2 and 4 years postoperatively.

This retrospective analysis is exempt from IRB review based on 45 CFR 46, “Collection or Study of Existing Data”, considering the HIPPA Privacy Rule (45 CFR 160 and 164a); this has been confirmed by the IRB at Providence Hospital in Columbia, SC.

## 3. Results

Group 1 comprised 1496 consecutive HRAs in 1276 patients performed between September 2006 and November 2011. After the wedge-fit method was fully implemented, the primary surgeon performed 1565 consecutive uncemented Biomet HRAs in 1365 patients prior to June 2016. The minimum follow-up period for both groups was 2 years, with an average of 4.7 years for Group 1 and 2.4 years for Group 2.

Failures due to lack of bone ingrowth into the acetabular component (Table 2) were significantly reduced after employing the wedge-fit approach (0.5% to <0.1%, p=0.03). Diagnoses were confirmed intraoperatively at revision surgery. With standard acetabular implantation techniques (Group 1), there was a 0.6% rate of revision due to early acetabular failures (before 2 years postoperatively); after the new bone preparation techniques, this reduced to 0.1% (p=0.03). The incidence of unexplained pain between 2 and 4 years declined significantly from 2.6% to 1.3% (p=0.006). Unrevised early cup shifts also reduced significantly from 1.1% to 0.4% (p=0.04).

While all patients were at least two years out from surgery, the rate of follow-up was significantly lower for Group 2 (Table 3). Of those that returned for their 2-year follow-up, mean Harris pain score and worse VAS pain score were similar, while regular VAS pain score was significantly higher for Group 1. UCLA score and combined range-of-motion were also similar between the two groups. There were no instances of radiolucency or osteolysis.

## 4. Discussion

Our data indicate that a wedge-fit acetabular preparation strategy promotes acetabular component stability and ensures a higher rate of stable bone ingrowth. We developed this wedge-fit strategy based on the hypothesis that failure of acetabular component ingrowth was due to inadequate initial fixation. The three prerequisites [19, 20] required to achieve bone ingrowth in uncemented implants include live and healthy bone, an adequate porous surface, and a tight initial fit that prevents micromotion of more than 100 *μ*m. If these conditions are not met, failure of bone ingrowth can occur. In our experience, failure of acetabular bone ingrowth most often presents in one of two ways. Either the component suddenly spins out, or the patient presents with explained pain and normal early radiographs. Over time, the component moves on serial x-rays, at which point the surgeon makes the diagnosis. There may be a third group of bone ingrowth failure in which the patient presents unexplained pain without any x-ray changes over time. The senior author (TPG) has rarely found failure of acetabular component ingrowth to be the explanation for previously unexplained pain. Instead, unexplained pain likely comes from soft tissue problems, back pain, or other unrelated issues.

With the previous standard reaming technique (no apex relief), the surgeon had less control over the implant contact points. We believed that, in some cases, the implant bottomed out on the apex with relative loose fit around the periphery. With eccentric loading, toggling of the implant could cause micromotion resulting in fibrous rather than bone ingrowth. We hypothesized that if we tailored the reaming method by bone density and relieved the apex, we could prevent apex toggling and achieve a more reliable press-fit with improved bone ingrowth.

Although Group 2 had a lower mean preoperative function score, clinical outcomes were largely similar. While Harris pain score and worst VAS pain score were similar between the two groups, mean regular VAS pain score was lower for Group 2. All Group 2 patients met the RAIL; this was significantly higher than Group 1. Likewise, average AIA was lower for Group 2 even though mean component size was similar between both cohorts.

There are some notable limitations to this study. There were some minor demographic differences between the two groups; Group 2 cases were slightly older, on average, but we have never selected against patients based on age. Additionally, there were significantly fewer cases of osteoarthritis in Group 1 and more cases of posttrauma than in Group 2. However, proportions of high-risk diagnoses (dysplasia and osteonecrosis) were similar.

Next, the x-rays required to diagnose early cup shift were not part of our protocol until November 2007. Thus, we likely missed some cup shifts in Group 1. When we compare only cases after these x-rays, the rate of unrevised cup shift is even higher in Group 1, and the difference in rate of shift between Groups 1 and 2 becomes even greater (p=0.009).

Another limitation is the difference in rate of follow-up and postoperative time. Group 1 had a longer follow-up interval and had more patients return for follow-up. However, when prompted, many patients that never returned for follow-up reported great results and felt no need to return. Therefore, it is unlikely that there were enough missed complications in Group 2 to change the results.

Lastly, this study investigates the outcomes of the wedge-fit method on the Biomet Magnum-ReCap™ resurfacing system only. While it seems possible that many hemispherical, porous-coated metal cups would behave similarly with these techniques, we cannot be certain. Titanium shells typically used in THA may be more flexible than the cobalt-chrome Magnum resurfacing component we evaluated, but all metal cups are much stiffer than bone and therefore may act similarly. The roughness of the porous coating and any supplemental fixation, such as screws or spikes, would likely markedly alter the results.

Additionally, an experienced resurfacing surgeon performed these cases. Reports suggest that HRA requires a steep learning curve [21]. Therefore, we cannot guarantee another surgeon employing the same device and method would achieve comparable results.

It is not clear whether more rigid cobalt-chrome resurfacing components have a higher rate of ingrowth failure than THA cups. According to New Zealand registry data [22], failure before 90 days postoperatively due to loose acetabular component was <0.1% for cemented, uncemented, and hybrid cemented THAs. However, we define failure of ingrowth as a loose acetabular component before 2 years postoperatively, so this rate in the New Zealand registry is not comparable. Latteier* et al.* [23] published outcomes for MoM THA and reported a higher incidence of failure of ingrowth (2.6%) than our study sample but is comparable with other reports on HRA. Kim* et al.* [24] published a multicenter study on HRA with a significantly higher rate of failure ingrowth at 5% for the Conserve Plus® cobalt-chrome cementless acetabular components. This study comprised 5 surgeons, only one of which was an experienced HRA surgeon. We have experienced a much lower rate of overall failure and of ingrowth failure in HRA. A detailed analysis of the Conserve Plus by Amstutz* et al*. [25] identified femoral loosening as the main mode of failure, with 1.8% of cases failing due to loosening. It is unclear how many of these were early loosenings. This compares to our rate of ingrowth failure prior to the wedge-fit technique.

## 5. Conclusions

After implementing the wedge-fit acetabular bone preparation method, we observed a reduction in the rate of failure of acetabular component ingrowth (0.5% versus <0.1%), the rate of asymptomatic cup shifts (1.1% versus 0.4%), and the incidence of unexplained pain between 2 and 4 years of follow-up (2.6% versus 1.3%). We suggest that some cases of unexplained pain may be the result of fibrous ingrowth into the acetabular component. Unfortunately, there is currently no reliable way to diagnose fibrous ingrowth except by exploring and removing an acetabular component, which could lead to unnecessary removal of a well-fixed cup.

## Figures and Tables

**Table 1:** Demographics.

Variable	Group 1	Group 2	P value
	(Before protocol)	(Wedge fit)	
Date Range	9/2006-11/2011	6/2012-6/2016	- -

# of Cases	1496	1565	- -

# Deceased*∗*	7 (0.5%)	3 (0.2%)	0.1802

Demographics	- -		

#, % Female	383 (25.5%)	415 (26.5%)	0.5619

Mean Follow-Up (Years)	4.7 ± 2.3	2.4 ± 1.3	<0.0001*∗*

Age (Years)	52.4 ± 8.3	54.2 ± 8.6	<0.0001*∗*

BMI	27.6 ± 4.5	27.8 ± 4.8	0.2349

T-Score	0.0 ± 1.3	0.0 ± 1.2	1.000

Femoral Component Size (mm)	49.9 ± 3.5	49.9 ± 3.6	1.000

Diagnoses	- -		

Osteoarthritis	1138 (76.1%)	1276 (81.5%)	0.0002*∗*

Dysplasia	198 (13.2%)	184 (11.8%)	0.2150

Osteonecrosis	76 (5.1%)	70 (4.5%)	0.4295

RA	6 (0.4%)	3 (0.2%)	0.2846

Post-Trauma	33 (2.2%)	17 (1.1%)	0.0147*∗*

LCP/SCFE	31 (2.1%)	26 (1.7%)	0.4009

Other	14 (0.9%)	15 (1.0%)	0.9522

**Table 2:** Failures and complications.

Failure Type	Group 1	Group 2	P value
# Cases	1496	1565	- -

(1) Revisions			

(a) Failure of Acetabular Ingrowth (<2 years)	7 (0.5%)	1 (<0.1%)	0.0271*∗*

(b) Adverse Wear	3 (0.2%)	0 (0.0%)	0.0735

(c) Late Acetabular Loosening (>2 years)	2 (0.1%)	0 (0.0%)	0.1443

(d) Acetabular Component Shift	1 (<0.1%)	1 (<0.1%)	0.9681

(2) Unrevised Cup shift	16 (1.1%)	7 (0.4%)	0.0424*∗*

(3a) Unexplained pain (>2 years, latest F/U)	31 (2.2%)	21 (2.6%)	0.1031

(3b) Unexplained pain (2-4 years, peak pain)	39 (2.6%)	20 (1.3%)	0.0061*∗*

**Table 3:** Clinical outcomes.

Variable	Group 1	Group 2	P value
Preoperative			

HHS Score	58.6 ± 13.2	57.3 ± 18.7	<0.0001*∗*

Postoperative			

Cases with 2-year FU (#, %)	1407 (94.1%)	1121 (71.6%)	<0.0001*∗*

Cases with any FU	1485 (99.3%)	1496 (95.6%)	<0.0001*∗*

HHS Score	98.1 ± 6.0	97.7 ± 6.2	0.1716

Harris Pain Score	42.8 ± 4.2	42.7 ± 4.1	0.6182

UCLA Score	7.6 ± 1.9	7.5 ± 1.9	0.3059

VAS^2^ Pain: Regular	0.3 ± 1.0	0.2 ± 0.9	0.0036*∗*

VAS Pain: Worse	1.2 ± 1.9	1.3 ± 2.0	0.2826

Combined ROM	272.2 ± 42.1	270.4 ± 34.4	0.5600

Radiographic Data			

AIA	35.9 ± 5.5	33.9 ± 4.9	<0.0001*∗*

Under RAIL (# Hips, %)	1266/1374 (92.1%)	1565/1565 (100.0%)	<0.0001*∗*

Radiolucency (# Hips, %)	0 (0.0%)	0 (0.0%)	1.000

Osteolysis (# Hips, %)	0 (0.0%)	0 (0.0%)	1.000

## Data Availability

Raw data is available upon request. Please contact Dani M. Gaillard-Campbell at dani.gaillard@midorthoneuro.com.

## Abbreviations

HRA: Hip resurfacing arthroplasty
THA: Total hip arthroplasty
MoM: Metal-on-metal
AWRF: Adverse wear-related failure
OD: Outer diameter
AIA: Acetabular inclination angle.
